# Identification of More Than Two Paternal Haplotypes of the Ovine Fatty Acid-Binding Protein 4 (FABP4) Gene in Half-Sib Families: Evidence of Intragenic Meiotic Recombination

**DOI:** 10.1371/journal.pone.0088691

**Published:** 2014-02-11

**Authors:** Wei Yan, Huitong Zhou, Yuzhu Luo, Jiang Hu, Jon G. H. Hickford

**Affiliations:** 1 Gansu Key Laboratory of Herbivorous Animal Biotechnology, Faculty of Animal Science and Technology, Gansu Agricultural University, Lanzhou, China; 2 Gene-Marker Laboratory, Faculty of Agriculture and Life Sciences, Lincoln University, Lincoln, New Zealand; University of Science and Technology of China, China

## Abstract

The fatty acid binding protein 4 (FABP4) plays an important role in the regulation of lipid metabolism in mammals. In this study, two regions of ovine *FABP4* spanning exon 2-intron 2 and exon 3-intron 3 were investigated in four hundred and twenty lambs derived from seven sires that were previously typed as having heterozygous genotypes in both these regions of the gene. These regions have been shown to be variable, with three SNPs plus one indel and four SNPs respectively constituting five and four allele variants in the two regions. Across these regions, fourteen haplotypes have been identified. The lambs were typed using a Polymerase Chain Reaction Single-Stranded Conformational Polymorphism (PCR-SSCP) method to identify the haplotypes inherited from the sires. Between three and four paternally-derived haplotypes were identified in the progeny of six of the seven sires, suggesting that meiotic recombination occurs within ovine *FABP4*. A number of sequence motifs associated with recombination “hotspots” were detected in the two regions of the gene that were analyzed and these may facilitate the recombination.

## Introduction

The fatty acid binding protein 4 (FABP4; also known as adipocyte fatty-acid binding protein) belongs to a fatty-acid binding protein family that is comprised of at least nine members in mammals [1]. The protein is predominantly expressed in white and brown adipose cells; but it is also found in many other cells and tissues, including blood, liver, heart, brain, skin and muscle [2]–[4]. As an intracellular protein, it is thought to be involved in lipid metabolism and acts by binding and transporting long-chain fatty acids [5].

In humans, the FABP4 gene has been shown to be associated with the risk of acquiring hyper-triglyceridemia, type 2 diabetes and cardiovascular disease [6]. Studies of farm animals have revealed that it may have an impact on growth in pigs [7], fat deposition, marbling and carcass weight in cattle [8], [9], and fleece-rot resistance in sheep [10].

*FABP4* consists of four small coding exons and three introns. The gene has been described in many species, but genetic variation has only been reported in some [6], [8], [11]. An unexpectedly high level of polymorphism has been reported in pigs, with approximately one nucleotide substitution in every 50 bp [7]. Little is known of the mechanism(s) that create(s) this genetic diversity.

**Figure 1:**
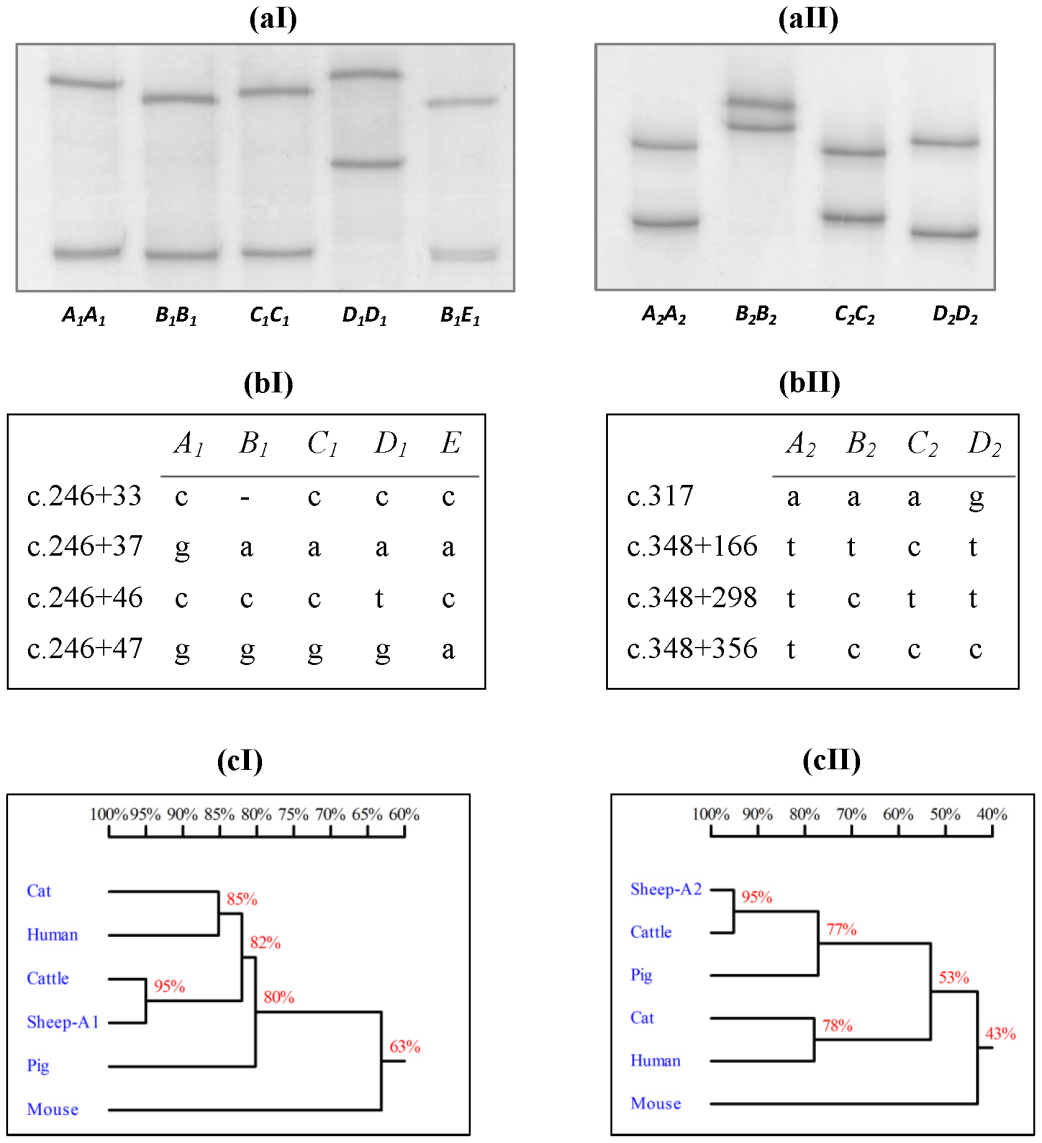
Polymorphism of ovine *FABP4* and sequence homology with other species. Five and four allele variants are respectively identified in region I (exon 2-intron 2) and region II (exon 3-intron 3) by (**a**) PCR-SSCP and (**b**) DNA sequencing [11]. The *FABP4* sequences from other species used to construct the homology trees (c) are: NC_007312 (cattle), EF061481 (pig), HQ384160 (human), ENSFCAG00000032028 (cat) and ENSMUSG00000062515 (mouse). The nucleotide numbering follows the nomenclature described on www.hgvs.org/mutnomen/.

We have recently reported five and four sequence variants, containing three SNPs plus one indel and four SNPs respectively, in two separate regions of ovine *FABP4* (Figure 1) [11]. Fourteen haplotype sequences were constructed across these two regions [11] and some of them appear to have been created by intragenic recombination events. These regions contain the majority of the *FABP4* coding sequence and some of the encoded amino acids appear to be important for the functional domains of the protein in the tertiary structure [12]. Sequence similarity is observed between species in these regions (Figure 1).

To confirm whether meiotic recombination occurs within the gene, we investigated the inheritance of paternal haplotypes in 359 lambs derived from seven sires.

## Materials and Methods

### Sheep blood and DNA samples

Blood samples from commercially farmed sheep on private farms were collected onto FTA cards (Whatman, Middlesex, UK) by the owners. The collection of blood drops by nicking sheep ears, is covered by Section 7.5 Animal Identification and Minimal Standard No. 13 - Identification, of the Animal Welfare (Sheep and Beef Cattle) Code of Welfare 2010; a code of welfare issued under the Animal Welfare Act 1999: Public Act 1999 No. 142, New Zealand (NZ) Government. Blood samples are routinely collected for commercial DNA testing for various traits.

Blood samples were collected from all the sheep onto FTA cards (Whatman, Middlesex, UK) and genomic DNA was purified using a two-step procedure [13]. Initially, by typing NZ Romney sires, seven were identified that were heterozygous in both regions of *FABP4*. These sires produced 420 lambs that were subsequently typed for two regions of *FABP4*. The lambs and sires were also typed for variation in *PRNP* [14], *ADRB3* [15] and the *MHC-DQA2 - DQA2-like* region [16] to confirm their sire group. The sires and lambs were all derived from the ongoing NZ Romney progeny test 2006-2013. This genetic trial involves the single-sire mating of selected NZ Romney rams to approximate 60 ewes each, with lambs identified to ram and ewe at birth and phenotypic data collected for growth and carcass traits. DNA is collected from all the sheep and lambs annually.

### Genotyping of ovine FABP4

Two regions of ovine *FABP4* were separately typed using a Polymerase Chain Reaction Single-Stranded Conformational Polymorphism (PCR-SSCP) method as described previously [11]. Briefly, region 1 (exon 2 - intron 2) was amplified using the PCR primers 5′-tgtgggctttgctaccag-3′ and 5′-taaatgggagacaattcacc-3′, while region 2 (exon 3 - intron 3) was amplified using the primers 5′-acttagatgaaggtgctctg-3′ and 5′- ctcaggactaaacaactcatg-3′. Amplifications were performed in a 15-µL reaction containing the DNA on one 1.2 mm punch of FTA card, 0.25 µM of each paired primer set, 150 µM dNTPs (Bioline, London, UK), 2.5 mM Mg^2+^, 0.5 U Taq DNA polymerase (Qiagen, Hilden, Germany) and 1× the reaction buffer supplied with the enzyme. The thermal profiles consisted of 2 min at 94°C, followed by 35 cycles of 30 s at 94°C, 30 s at 60°C and 30 s at 72°C, with a final extension of 5 min at 72°C.

After denaturation and rapid cooling on wet ice, PCR amplicons were electrophoresed in 14% (for region 1), or 10% (for region 2) polyacrylamide gels for 19 hours in 0.5×TBE at 7.5°C and 390 V (for region 1) or 300 V (for region 2). Amplicons of the known variant sequences [11] were included in each polyacrylamide gel and their banding patterns were used as references for determining the genotypes of individual progeny. Gels were silver-stained according to the method of Byun et al. [17].

### Determination of paternal haplotypes

At each region, by comparing the progeny and sire genotypes, the sequence variants inherited from the sires could be determined for the progeny. The exception was with lambs that had the same genotypes as their sires. These lambs were excluded from analysis as the variants inherited from the sires could not be resolved, and maternal DNA was not available to assist in this resolution.

Having determined the sequence variants inherited from the sire for each of the two regions, the paternal haplotypes across the regions could be constructed.

## Results

Of the 420 progeny investigated, 188 (44.8%) possessed the same genotypes as their sires at either one or both regions of *FABP4*, and hence the haplotypes inherited from the sires across these regions could not be determined. For the remaining 232 (55.2%) of the progeny, the parental haplotypes could be determined. More than two paternal haplotypes were identified in all but one of the sire-lines (Table 1). In each of the sire-lines where more than two paternal haplotypes were observed, only two haplotypes were common, while the other/others was/were detected at a lower frequency (Table 1). These less frequent haplotype(s) could be generated from the main paternal haplotypes by meiotic DNA recombination between the two gene regions that were amplified.

**Table 1:** Paternal haplotypes of ovine *FABP4* detected in individual sire-lines.

Sire ID	Sire genotype		Total number of progeny	Progeny with paternal haplotype identified	
	Region 1	Region 2		Paternal haplotype	Number (frequency)
Totaranui 376/02	*A_1_/D_1_*	*A_2_/B_2_*	67	*A_1_-B_2_*	23 (57.5%)
				*D_1_-A_2_*	17 (42.5%)
Waidale 618/04	*C_1_/D_1_*	*A_2_/C_2_*	68	*C_1_-C_2_*	22 (46.8%)
				*D_1_-A_2_*	24 (51.1%)
				*C_1_-A_2_* *	1 (2.1%)
Snowlea 192/02	*B_1_/C_1_*	*B_2_/C_2_*	65	*B_1_-B_2_*	16 (45.7%)
				*C_1_-C_2_*	18 (51.4%)
				*C_1_-B_2_* *	1 (2.9%)
Mana 83/04	*B_1_/C_1_*	*B_2_/C_2_*	62	*B_1_-B_2_*	17 (54.8%)
				*C_1_-C_2_*	13 (41.9%)
				*C_1_-B_2_* *	1 (3.2%)
Doughboy 45/04	*B_1_/C_1_*	*A_2_/B_2_*	69	*B_1_-B_2_*	22 (52.4%)
				*C_1_-A_2_*	16 (38.1%)
				*C_1_-B_2_* *	4 (9.5%)
Glenleith 25/02	*A_1_/B_1_*	*A_2_/B_2_*	42	*A_1_-A_2_*	11 (57.9%)
				*B_1_-B_2_*	7 (36.8%)
				*B_1_-A_2_* *	1 (5.3%)
Mana 90/01	*A1/C1*	*A_2_/B_2_*	47	*A_1_-B_2_*	8 (44.4%)
				*C_1_-A_2_*	7 (38.9%)
				*A_1_-A_2_* *	2 (11.1%)
				*C_1_-B_2_* *	1 (5.6%)

* Recombined minor sire haplotypes.

The paternity of all the lambs was confirmed by typing for variation in *PRNP*, *ADRB* and the *MHC-DQA2 - DQA2-like* region (results not shown).

**Figure 2:**
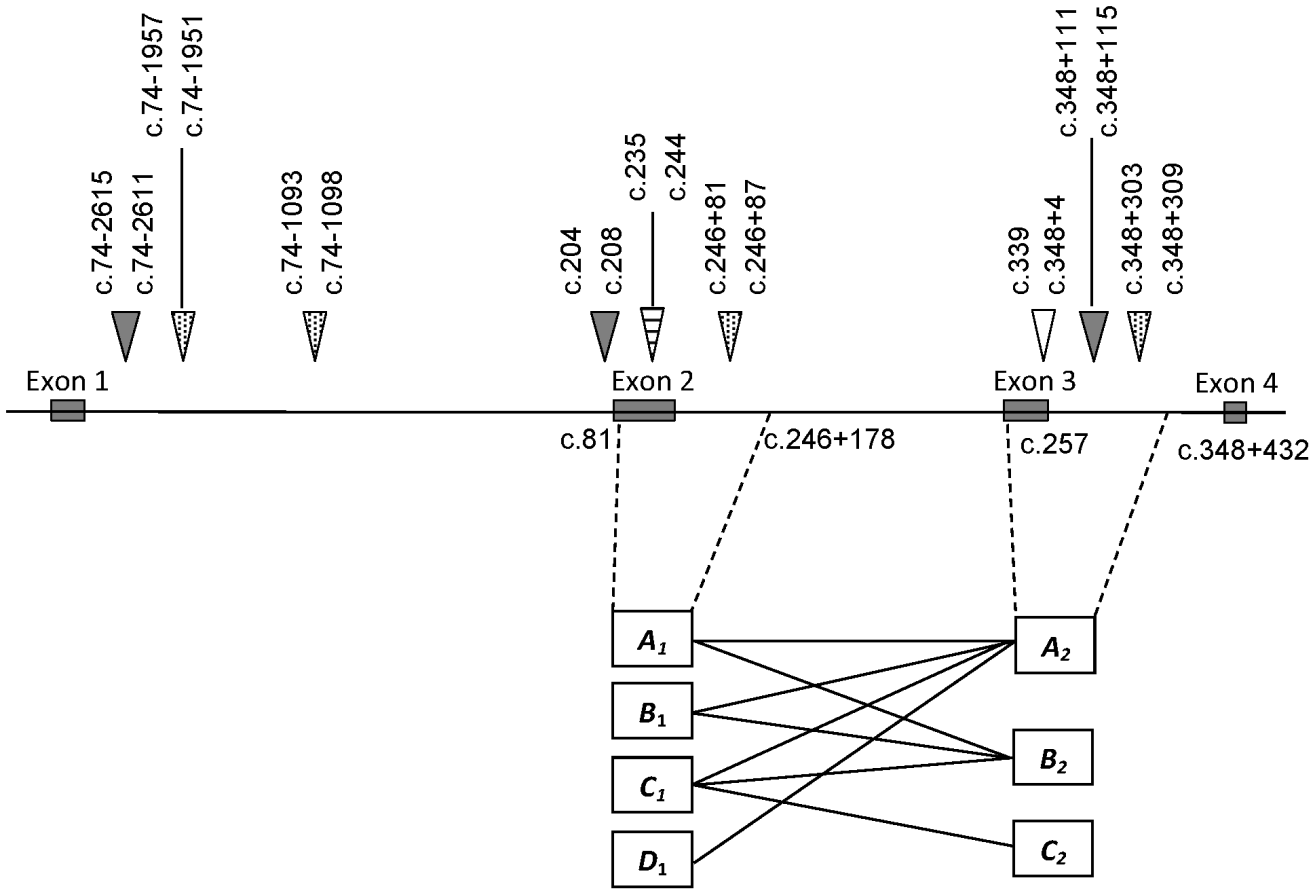
The presence of potential “recombination hotspots” in ovine *FABP4* and the haplotypes across two gene regions that were inherited from the seven NZ Romney sires. The locations of these two regions in ovine *FABP4* are indicated. An unfilled triangle represents uble overlapping *chi*-like sequences (GCTGGTGCTGGTGA), and a horizontal line filled triangle represents the *CRE*-like sequence (ATGAAGTCA). The CCTCCCT motifs and variants are indicated by dot filled triangles, and the CCAAT motifs are shown as filled triangles. The numbering of nucleotide positions follows the nomenclature described at www.hgvs.org/mutnomen/

Within the ovine *FABP4* regions analysed, we found a number of simple sequence motifs that have been reported to be associated with recombination hotspots in both prokaryotes and eukaryotes. These included: 1) a 14-mer sequence (GCTGGTGCTGGTGA) consisting of two partially overlapping *chi*-like sequences (GCTGGTGC and GCTGGTGA) in region 2; 2) a *CRE*-like sequence (ATGAAGTCA) in region 1; 3) a CCTCCCT motif approximately 2 kb upstream of region 1 and variants of this sequence in region 1 and region 2; and 4) a number of copies of a CCAAT motif in region 1, region 2, and other regions of the gene (Figure 2).

## Discussion

In this study, we report the detection of three or four haplotypes of ovine *FABP4* inherited from a single sire. Of these, two haplotypes were commonly found, and the less common haplotypes appear to have come-about from meiotic recombination between the two regions of the gene that were amplified. In the absence of meiotic recombination, only two sire haplotypes would typically be observed, whereas theoretically four different paternal haplotypes including two un-rearranged and two recombined haplotypes would be expected in the progeny if a single recombination event occurred within a gene. If we assume that the rate of recombination is the same across all the sires, then based on the recombination that could be observed in the 232 typeable progeny, we would guess the average recombination rate is 11/232 genotypes or 4.7%. This is considerably higher than one might expect given the proximity of the two regions typed in this gene, but caution is needed in making this interpretation given that only 232 out of 420 progeny could be typed.

The detection of only one recombined haplotype in some sire-lines may be because the paternal haplotypes in approximately half of the progeny could not be determined using the PCR-SSCP technique (the progeny typed the same as the sire) and/or because the recombined haplotypes occurred at a low frequency. It seems likely that other recombined paternal haplotypes could be detected if all the progeny could be effectively typed or additional sire lines were typed.

Meiotic recombination occurs frequently in some regions of various genomes and these have been called “recombination hotspots” [18]. The presence of a number of motifs that have been associated with “recombination hotspots” in ovine *FABP4* (Figure 2) supports our contention that meiotic recombination occurs with this gene.

Firstly two partially overlapping *chi*-like sequences (GCTGGTGC and GCTGGTGA) were found in region 2 of the gene. The *chi* sequence, GCTGGTGG, is the most notable hotspot known to enhance homologous recombination in *E. coli* [19]. It serves as a stimulator of DNA double-strand break repair in bacteria, and the resulting single-stranded DNA (ssDNA) is bound by multiple molecules of RecA protein that facilitate “strand invasion” in which one strand of a homologous double-stranded DNA is displaced by the RecA-associated ssDNA [20]. A homolog of bacterial RecA exists in eukaryotes including mice and humans [21], [22]. The presence of two *chi*-like sequences in ovine *FABP4* may be important for DNA recombination, as *chi*-like sequences may be implicated in the activity of recombination hotspots [23]. It is interesting that these two *chi*-like sequences are partially overlapped in a 14-mer sequence GCTGGTGCTGGTGA, and that the presence of a C at the eighth position of the 14-mer motif eliminates a *chi* site, but creates two partially over-lapping *chi*-like sequences. This 14-mer motif has been previously observed for recombined *fimA* sequences from *Dichelobacter nodosus* [24].

A *CRE-*like sequence (ATGAAGTCA) was found in region 1 of the gene. *The CRE* sequence (ATGACGTCA), also known as *M26*, is the binding site for a hetero-dimeric transcription factor Aff1-Pcr1, and it acts as a recombination hotspot in the yeast *Schizosaccharomyces pombe* (*S. pombe*) [25], [26].

Thirdly, the presence of a CCTCCCT motif and variants of this motif in both regions of the gene may promote recombination. In humans, the CCTCCCT motif is present more frequently in recombination hotspots than elsewhere, and chromosomes active for recombination contain this motif, with the “suppressor” mutation being a change from T to C in its third position [27].

Lastly repeats of a CCAAT motif were present in the gene. The CCAAT motif appears to be associated with recombination hotspot activity in *S. pombe* [28].

It is also notable that the human FABP4 gene sequence GenBank accession number HQ384160, appears to contain an ALU-like sequence in intron 2 (nucleotides 5801-6089). Ovine intron 2 (accession number NC_019466) and human intron 2 (accession number HQ384160 share approximately 38% homology (results not shown), a major difference being that the ovine intron is much shorter and does not contain a region comparable to the ALU-like sequence. ALU sequences are proposed to facilitate local recombination [29], but the absence of this sequence in sheep, would suggest it is not responsible for the observed recombination.

The presence of these potential “recombination hotspots” in ovine *FABP4* supports the contention that intragenic recombination has occurred in this gene. This is consistent with our previous finding that five sequence variants in region 1 and four variants in region 2 generate 14 different haplotypes across these two regions of the ovine FABP4 gene [11].
